# Effect of breeding performance on the distribution and activity budgets of a predominantly resident population of black‐browed albatrosses

**DOI:** 10.1002/ece3.5416

**Published:** 2019-07-17

**Authors:** Aurore Ponchon, Thomas Cornulier, April Hedd, José Pedro Granadeiro, Paulo Catry

**Affiliations:** ^1^ MARE, Marine and Environmental Sciences Centre ISPA – Insituto Universitário Lisboa Portugal; ^2^ School of Biological Sciences University of Aberdeen Aberdeen UK; ^3^ Psychology Department Memorial University of Newfoundland St. John's Newfoundland and Labrador Canada; ^4^ Environment and Climate Change Canada Mount Pearl Newfoundland and Labrador Canada; ^5^ Departamento de Biologia Animal, Faculdade de Ciências CESAM, Universidade de Lisboa Lisboa Portugal

**Keywords:** active foraging, breeding failure, carry‐over effects, migration, reproductive cost, sustained flight, wintering grounds

## Abstract

Pelagic seabirds breeding at high latitudes generally split their annual cycle between reproduction, migration, and wintering. During the breeding season, they are constrained in their foraging range due to reproduction while during winter months, and they often undertake long‐distance migrations. Black‐browed albatrosses (*Thalassarche melanophris*) nesting in the Falkland archipelago remain within 700 km from their breeding colonies all year‐round and can therefore be considered as resident. Accordingly, at‐sea activity patterns are expected to be adjusted to the absence of migration. Likewise, breeding performance is expected to affect foraging, flying, and floating activities, as failed individuals are relieved from reproduction earlier than successful ones. Using geolocators coupled with a saltwater immersion sensor, we detailed the spatial distribution and temporal dynamics of at‐sea activity budgets of successful and failed breeding black‐browed albatrosses nesting in New Island, Falklands archipelago, over the breeding and subsequent nonbreeding season. The 90% monthly kernel distribution of failed and successful breeders suggested no spatial segregation. Both groups followed the same dynamics of foraging effort both during daylight and darkness all year, except during chick‐rearing, when successful breeders foraged more intensively. Failed and successful breeders started decreasing flying activities during daylight at the same time, 2–3 weeks after hatching period, but failed breeders reached their maximum floating activity during late chick‐rearing, 2 months before successful breeders. Moon cycle had a significant effect on activity budgets during darkness, with individuals generally more active during full moon. Our results highlight that successful breeders buffer potential reproductive costs during the nonbreeding season, and this provides a better understanding of how individuals adjust their spatial distribution and activity budgets according to their breeding performance in absence of migration.

## 1 INTRODUCTION

During their lifetime, animals have to allocate time and energy to several competing activities such as foraging, breeding, resting, molting, or migrating (Stearns, 1992). Therefore, they are expected to adopt an optimal allocation strategy minimizing energetic costs and maximizing fitness, especially when they face adverse environmental conditions and energetic constraints (Williams, 1966).

Seabirds are of particular interest when studying resource allocation trade‐offs because they spend most of their life at sea but have to come back to land to breed. During the breeding season, seabirds are central place foragers: They feed at sea but have to regularly come back to their breeding site to ensure reproductive duties such as nest defense, egg incubation, and chick provisioning (Orians & Pearson, 1979). After the breeding season, seabirds are relieved from breeding constraints and most species leave their colony and migrate to reach distant, more favorable nonbreeding grounds. As long‐lived species, they also have to carefully balance their activity budgets between current breeding investment and self‐maintenance to maximize the survival probability of their young, without compromising their own survival (Williams, 1966). Their activity budgets are therefore expected to be adjusted accordingly to replenish energy reserves postbreeding, while minimizing energetic costs of migration.

Migration strategies are highly diverse between seabird species, but also within and between populations of the same species. For example, individual Cory's shearwaters *Calonectris borealis* breeding in Selvagem Grande (Madeira archipelago) spend their nonbreeding season in five different areas (Dias, Granadeiro, & Catry, 2012) while individuals from this species breeding in other colonies converge to the same nonbreeding grounds (González‐Solís, Croxall, Oro, & Ruiz, 2007). Likewise, individuals from some populations may migrate thousands of kilometers while others remain relatively sedentary (Pérez, Granadeiro, Dias, Alonso, & Catry, 2014; Ramos, Llabrés, Monclús, López‐Béjar, & González‐Solís, 2018; Weimerskirch, Delord, Guitteaud, Phillips, & Pinet, 2015). This is notably the case of the black‐browed albatross *Thalassarche melanophris*. This large procellariiform seabird breeds annually on several small subantarctic islands around the Southern Ocean. While populations nesting in South Georgia and Kerguelen undertake long‐distance migrations after the breeding season to reach areas situated thousands of kilometers away from their nesting area (Desprez, Jenouvrier, Barbraud, Delord, & Weimerskirch, 2018; Mackley et al., 2010; Phillips, Silk, Croxall, Afanasyev, & Bennett, 2005), black‐browed albatrosses breeding in the Falklands' archipelago remain close to the Patagonian shelf year‐round, displaying a nonbreeding range that largely overlaps with the foraging areas used during breeding (Grémillet, Wilson, Wanless, & Chater, 2000). Because of this residency, individuals are expected to specifically adjust their time spent foraging, flying, and floating on the water (hereafter called at‐sea activity budgets). In particular, they are expected to spend less time actively flying at the beginning of the nonbreeding season, as they do not have to invest in long‐distance migration.

Likewise, breeding performance is expected to affect both nonbreeding distribution and general activity during the subsequent nonbreeding season. Indeed, as failed breeders are relieved from reproductive duties earlier than individuals that successfully fledge a chick, they do not suffer from the same potential reproductive costs (Golet, Schmutz, Irons, & Estes, 2004; Ramos et al., 2018). Failed breeders have notably been shown to leave the colony earlier than successful breeders (Bogdanova et al., 2011; Catry, Dias, Phillips, & Granadeiro, 2013a; Desprez et al., 2018) and use different nonbreeding grounds (Bogdanova et al., 2011; Catry, Dias, et al., 2013a; Clay et al., 2016; Hoye, Hahn, Nolet, & Klaassen, 2012), which may directly influence their activity budgets, both at the end of the breeding season and during the subsequent nonbreeding season (Ramos et al., 2018). In migrant black‐browed albatrosses, failed breeders have notably been shown to forage more during the nonbreeding season (Desprez et al., 2018).

In this study, we examined the spatial distribution and temporal dynamics of at‐sea activity budgets of black‐browed albatrosses nesting in New Island, Falklands archipelago, from mid‐incubation until the end of the subsequent nonbreeding season, 11 months later. We compared distributions and activity budgets during day and night for successful (individuals that successfully raised a chick) and failed breeders (those that laid an egg that did not hatch or lost a young chick), while accounting for sex. We hypothesized that failed breeders had a larger and segregated nonbreeding distribution compared to successful breeders because they did not have to invest anymore in parental care and were relieved from central place foraging constraint earlier (Clay et al., 2016). Meanwhile, successful breeders were expected to spend more time foraging and flying during the chick‐rearing period, as they have to forage both for the chick and themselves. Moreover, as individuals do not undertake long‐distance migration, we expected an earlier decrease of flying activities in failed breeders, along with an increase of floating activities associated with resting phases, potentially before the end of the breeding season. Finally, as most albatrosses including black‐browed albatrosses are mainly diurnal (Catry, Ramos, Corre, & Phillips, 2009; Mackley et al., 2010; Phalan et al., 2007), we expected lower foraging and flying activities and higher floating activity at night, regardless of individual breeding success. Nevertheless, we expected an increase of foraging and flying activities during full moon compared to new moon (Dias et al., 2012; Hedd, Gales, & Brothers, 2001; Pinet, Jaeger, Cordier, Potin, & Corre, 2011; Yamamoto et al., 2008).

## 2 MATERIAL AND METHODS

### 2.1 Bird tracking

Fieldwork was carried out on New Island (51°51′S, 61°18′W), in the Falklands Archipelago, where *ca*. 12,000 black‐browed albatrosses breed each year. Leg‐mounted Mk7 or Mk19 light‐based geolocators including a saltwater immersion sensor (British Antarctic Survey) were deployed on 66 adult black‐browed albatrosses, all from different nests, during early incubation, in October 2012 and recovered the subsequent year, in November 2013. Of these loggers, 60 were successfully downloaded and provided exploitable data. Individuals were thereafter divided into two groups: successful breeders, which had successfully produced a chick (*n* = 48; 22 females and 26 males), and failed breeders, which had laid an egg but lost it before hatching or during chick‐rearing (*n* = 12; 6 males and 6 females). Individual laying and hatching dates were known for all individuals, as nests were monitored every 1–3 days from beginning of October 2012 to the end of February 2013. Sex was determined genetically by molecular procedures using DNA extracted from blood samples (Griffiths, Double, Orr, & Dawson, 1998) or from direct observation of nesting behavior during the prelaying period. The mean laying date was 11 October 2012 ± 2 days, the mean hatching date was 18 December 2012 ± 3 days, and the mean failure date was 26 December 2012 ± 29 days (see Table S1 for detailed individual data).

### 2.2 At‐sea distribution and activity budgets

Maximum light intensity levels, which were recorded every 5 (Mk19) or 10 min (Mk7), were decompressed using BASTrack software and then processed in MultiTrace Geolocation (Jensen Software Systems) using methods outlined in Phillips, Silk, Croxall, Afanasyev, and Briggs (2004). Using a light level threshold of 10 and a sun elevation angle of −3.5 (Mk19) or −4.0 (Mk7), individual positions were derived twice a day, with an expected accuracy of 186 ± 114 km (Phillips et al., 2004). Resulting locations were individually examined in ArcGIS by the same observer and unrealistic positions due to interferences in light curves at dawn and dusk and the 10 days before and after equinox periods were excluded from the analysis.

**Figure 1:**
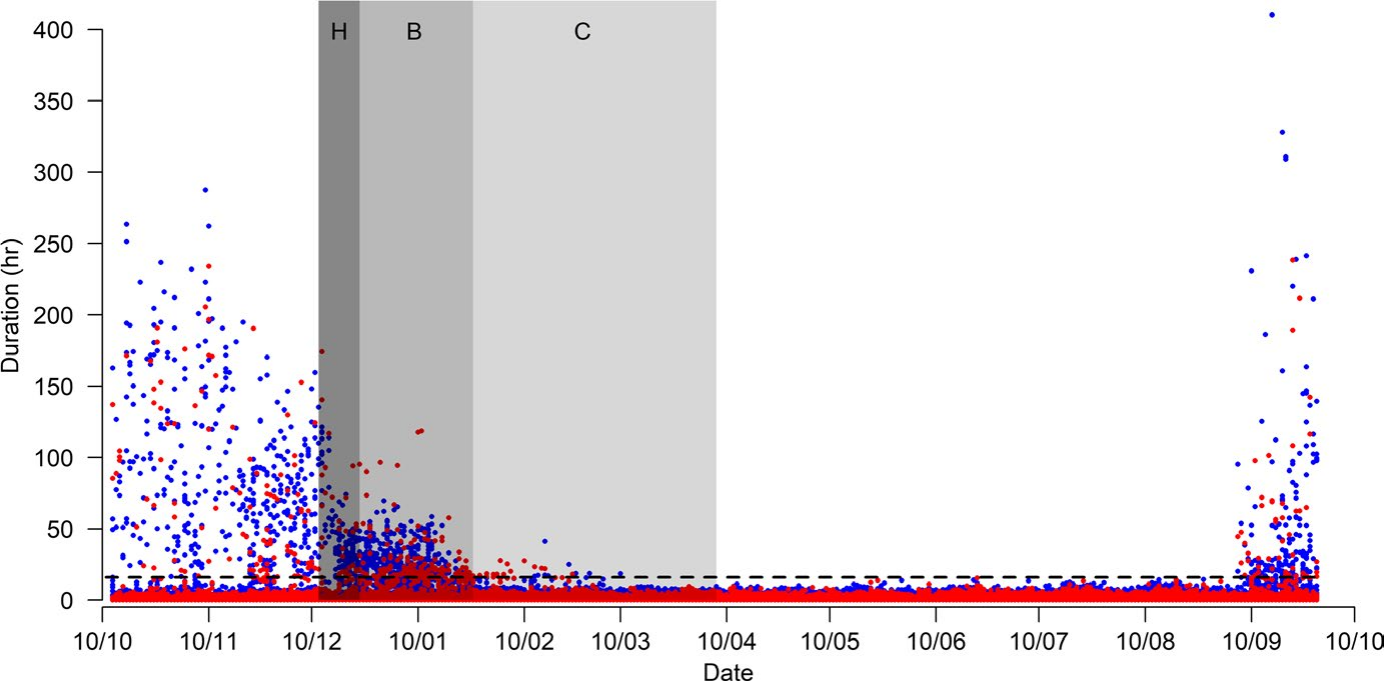
Raw activity data of dry events for successful (blue) and failed (red) black‐browed albatrosses. Dry events longer than the threshold duration of 16 hr (dashed horizontal line) were considered as periods on land, since they occurred only during the breeding season. Gray areas represent the different phases of breeding, from hatching (H) to brooding (B) and chick‐rearing period (C)

Three different activities were derived from the salt immersion sensor recording wet and dry state duration with a 3 s resolution: active foraging, sustained flight, and floating on the water. These activities were discriminated using the foraging bout interval criterion, a threshold duration delineating rapid wet–dry changes and longer wet or dry events. The foraging bout interval criterion was calculated for each individual using a maximum likelihood approach developed in the *diveMove* package (Luque, 2007) and was then averaged to obtain a single value of 53.16 min (see Dias et al., 2012 for further details on methodology). Thus, dry or wet periods shorter than 53.16 min were considered as foraging, dry periods longer than 53.16 min were considered as sustained flight, and wet periods longer than 53.16 min were considered as floating on the water. Immersion changes lasting less than 1.5 min were excluded to avoid the inclusion of short‐term variations in wet/dry status, which could be due to causes other than long events of foraging (Dias et al., 2012), as well as extensive dry periods >16 hr, likely corresponding to incubation and brood guarding shifts on land (Figure 1).

The daily percentage of time spent in each of the three activities was calculated separately for daylight and darkness, inferred from the dawn and dusk times obtained from the light data. As all our tracked individuals did not show clear shifts in latitude and/or longitude due to their annual residency, we could not delimit the end of the breeding season and beginning of the nonbreeding season at the individual level, when the birds completely leave the colony. Nevertheless, chick brooding has been estimated to last a maximum of 33 days in the colony of New Island (Catry et al., 2010), and the combined chick brooding and rearing period was 116 days from hatching in the colony of Bird Island, South Georgia (Tickell & Pinder, 1975). Moreover, raw activity data clearly showed a drop in the duration of long dry periods a few weeks after hatching and an absence of dry events longer than 16 hr after 10th March (Figure 1). Thus, we delimited three periods to characterize the breeding season based on the literature: hatching (12–24 December 2012), chick brooding during which parents still highly attended the nest and brood the chick (24 December 2012 to 26 January 2013), and chick‐rearing during which parents spend less time at the colony (26 January to 7 April 2013). Finally, based on the raw dry activity data, we assumed that the nonbreeding season ended on 5 September 2013, when very long dry activity data >16 hr likely corresponding to long periods on land were recorded again (Figure 1). Individual return dates were extracted based on this last criteria (*n* = 56).

### 2.3 Spatial data analysis

Individual monthly at‐sea distribution was inferred separately for successful and failed breeders from the calculation of the 50% and 90% utilization distribution (UD) contours using the “adehabitatHR” package (Calenge, 2006) with a cell size of 1,000 m and a smoothing factor of 100 km. The representativeness of the sample size for successful (*n* = 48) and failed breeders (*n* = 12) as well as males (*n* = 32) and females (*n* = 28) was assessed using a bootstrap analysis with 100 iterations, as detailed in Lascelles et al. (2016). The results were very similar between the 50% and 90% UD contours, so only results for the 90% UD contours are reported (Figure S1).

Spatial overlaps between the 50% and 90% UD of successful and failed breeders were estimated with a randomization procedure for each month separately, from February to September. Briefly, an initial spatial overlap matrix was calculated with the *kerneloverlap* function of the “adehabitatHR” package, using the Utilization Distribution Overlap Index (UDOI; Calenge, 2006) for each pair of individuals, regardless of their breeding performance. A second “membership” matrix indicating whether a pair of individuals had the same breeding performance (coded 0) or a different breeding performance (coded 1) was built. After removing diagonals from both matrices, a Pearson correlation coefficient *r*_obs_ was calculated between the two matrices. Then, the distribution of each individual was randomly and independently rotated around the colony location. A new spatial overlap matrix and a new membership matrix were built, and a new Pearson's correlation coefficient *r*_rand_ was calculated. This randomization procedure was repeated 1,000 times to obtain a distribution of correlation coefficient *r*_rand_ representing the null hypothesis of no difference in the spatial distribution of the two groups (see Cecere et al., 2018 for the detailed procedure). The same analysis was run between males and females.

Differences in the distribution of distance to the colony were assessed with bootstrap analyses based on 1,000 randomizations for sex and breeding status (interaction not significant). Breeding stages (breeding vs. nonbreeding period) were not compared because the exact date of the beginning of the nonbreeding season for individuals was unknown.

### 2.4 Activity data analysis

To explore the temporal dynamics of at‐sea activity over the breeding and subsequent nonbreeding season, we used generalized additive mixed models (GAMMs) with a quasi‐binomial error. Models were specified for the proportions of each activity and for daylight and darkness separately using the library *mgcv* in R (Wood, 2017). We included breeding status as a fixed categorical effect, and bird identity as a random intercept. The data did not support a sex effect in the trends, so this was excluded from the models. We included nonlinear smooth effects of the number of days since laying (calculated based on individual laying dates and covering the breeding as well as the subsequent nonbreeding period) as a thin‐plate spline with 12 knots, and moon cycle as a cyclic cubic spline with default number of knots (*k* = 3). In order to ensure that the effect of time since laying was estimated reliably, we had to account for the temporal nonindependence of data (pseudoreplication) within individual. This was done by decomposing the effect of days since laying into two group‐level smooth effects (respectively for failed and successful breeders, i.e., the focus of our analysis) and individual‐level smooth effects (“factor‐smooths”) which were designed to describe the departure of each individual from the group‐level effect and limit the risk of overfitting the group‐level effect. Smoothness parameter selection was performed using the default GCV method. For all activity proportions, the group‐level temporal trend was significantly different between successful and failed breeders. The significance of factor‐smooth interactions (i.e., an individual‐level smooth effect of days since laying) suggested clear interindividual variation around the respective mean temporal group trends. As expected, visual inspection of the group‐level smooth effects (the focus of our analyses) indicated that more complex trends were selected when individual‐level smooths were omitted from the model, suggesting that these were both supported and successful at limiting overfitting of the group‐level effects. Two alternative parameterizations of the factor‐smooth interactions were tested, one assuming identical smoothing level across individuals, and one where this assumption was relaxed. The first model presented a lack of identifiability between the population‐level and individual‐level terms (as evidenced by severe variance inflation of both terms, and nonstationarity in the mean of the factor‐smooths). As a consequence, the second parameterization with different smoothing parameters between individuals was chosen and presented here.

Moon cycle was obtained from the fraction of the moon illuminated at midnight for each day of the study period (20 October 2012–5 September 2013) provided by the United States Naval Meteorology and Oceanography Command website (https://aa.usno.navy.mil/data/docs/MoonFraction.php). It has to be noted that the quasi‐binomial distribution does not constrain the proportions of the three activities to sum to 1, but the difference was negligible in our case.

Daily proportions of time for each activity were further split over two periods covering one moon cycle to be compared: early chick‐rearing (28/01/2013 to 25/02/2013) and nonbreeding (24/06/2013 to 21/07/2013). We chose early chick‐rearing because flying activities might be slightly overestimated during the brooding period if some dry periods on land <16 hr are not correctly distinguished from flight at sea (Figure 1). We tested potential differences in the proportions of activities for sex, breeding status, their interaction, day period (daylight vs. darkness), and annual cycle (breeding vs. nonbreeding season) using bootstrap analyses on individuals based on 1,000 randomizations.

Data analyses were performed using R 3.4.1 (R Core Team, 2017). Results are shown as mean ± *SD* and *p*‐values <0.05 were considered to be statistically significant.

## 3 RESULTS

### 3.1 Spatial distribution

**Figure 2:**
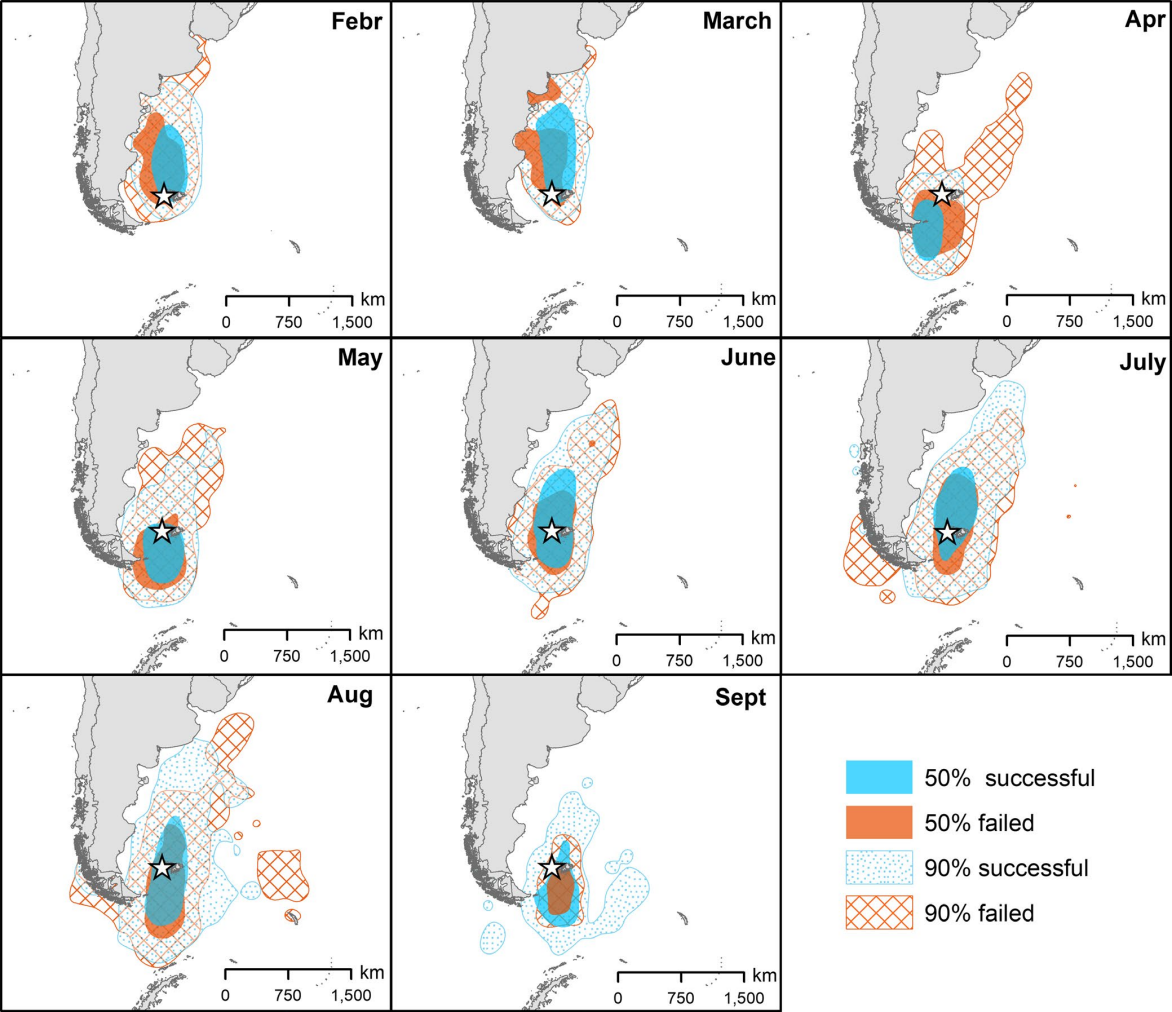
Monthly spatial distribution of successful (blue) and failed (red) breeding black‐browed albatrosses represented by 50% and 90% UD contours. The star represents the breeding colony of New Island. Note that distributions for March and September are only based on data from the first 10 days, as locations around equinoxes are unreliable

Representativeness of tracked individuals over the study period was very high (>94%; Figure S1), indicating that we captured most of the variability in space use by individuals over the study period. Moreover, the steep curves of the representativeness analysis indicated that birds belonging to same group showed highly overlapping home ranges. The 50% kernel density contours of both groups consistently included the colony, except in April (Figure 2). The randomization procedure further demonstrated that successful and failed breeders were not clearly spatially segregated over the study period, except for their 50% UD in February and August (*p* < 0.01; Table 1). Likewise, males and females were not clearly spatially segregated, except for their 90% UD in February (*p* = 0.04; Table 1). From February to the beginning of September, failed black‐browed albatrosses remained on average 560 ± 402 km away from their nesting colony, similarly to successful breeders which remained 505 ± 350 km away (Figure S2; Table S2). Nevertheless, females tended to remain further away from the colony than males (respectively 585 ± 413 km and 455 ± 294 km; Figure S2; Table S2). Although most of individuals remained in the Patagonian shelf all year, two successful and one failed breeders traveled around the tip of South America into the Pacific, off the coasts of Chile in July and August (Figure 2).

**Table 1:** Observed and randomized overlap (UDOI method) of 50% and 90% UDs between failed and successful breeders and between males and females

	50% UD			90% UD		
	*r*_obs_	*r*_rand_	*p*‐value	*r*_obs_	*r*_rand_	*p*‐value
Breeding performance						
February	−0.48	−0.38 ± 0.03	**0.002**	0.45	0.39 ± 0.02	0.07
March	−0.12	−0.10 ± 0.03	0.23	0.13	0.11 ± 0.02	0.21
April	−0.11	−0.09 ± 0.02	0.09	−0.14	−0.11 ± 0.02	0.08
May	−0.06	−0.03 ± 0.02	0.06	0.06	0.06 ± 0.01	0.28
June	−0.009	0.008 ± 0.02	0.20	0.005	0.01 ± 0.01	0.37
July	−0.04	−0.02 ± 0.02	0.22	0.03	−0.04 ± 0.01	0.39
August	−0.07	−0.02 ± 0.02	**0.01**	0.05	−0.04 ± 0.02	0.09
September	0.13	0.11 ± 0.05	0.61	0.10	0.10 ± 0.04	0.48
Sex						
February	−0.02	−0.005 ± 0.01	0.06	−0.03	−0.009 ± 0.008	**0.04**
March	−0.02	−0.03 ± 0.02	0.73	−0.05	−0.04 ± 0.02	0.38
April	−0.02	−0.03 ± 0.01	0.80	−0.03	−0.04 ± 0.007	0.70
May	−0.03	−0.04 ± 0.01	0.66	−0.05	−0.05 ± 0.006	0.54
June	−0.09	−0.09 ± 0.02	0.58	−0.11	−0.11 ± 0.01	0.48
July	−0.03	−0.05 ± 0.02	0.95	−0.08	−0.07 ± 0.008	0.23
August	−0.04	−0.07 ± 0.1	0.99	−0.09	−0.10 ± 0.010	0.72
September	−0.10	−0.10 ± 0.05	0.50	−0.16	−0.15 ± 0.04	0.39

Note

Randomized correlation coefficients are shown as mean ± *SD* and *p*‐values represent the proportion of randomized correlation coefficients that were lower than the observed one. Significant spatial segregations are shown in bold.

### 3.2 Temporal dynamics of activity budgets

**Figure 3:**
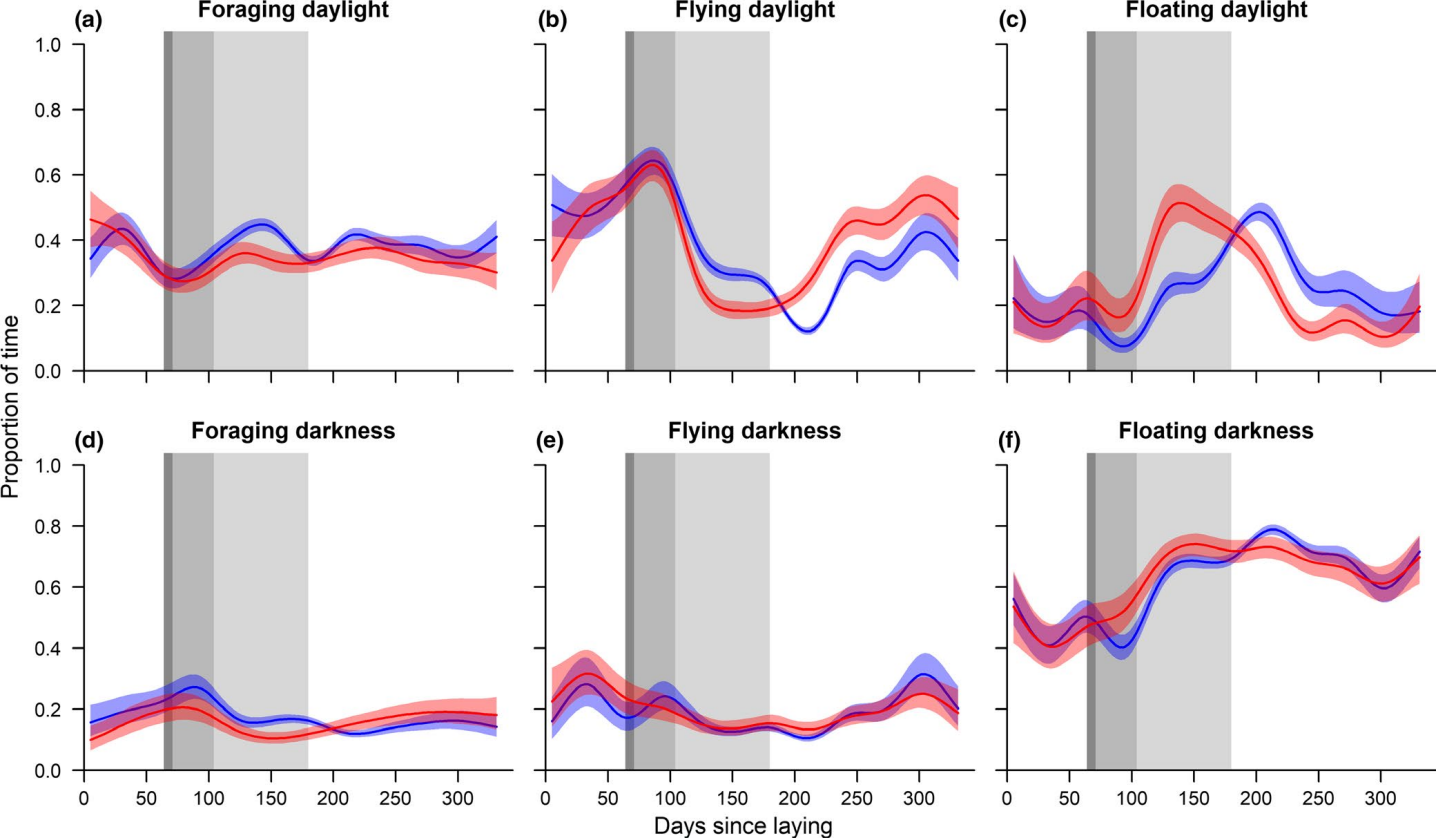
Temporal dynamics of the proportion of time spent foraging, flying, and floating on the water over the annual cycle of failed (red) and successful breeders (blue) during daylight (upper panel) and darkness periods (lower panel). Colored shaded areas represent 95% confidence intervals while dark to light gray areas represent hatching, chick brooding, and chick‐rearing period

**Figure 4:**
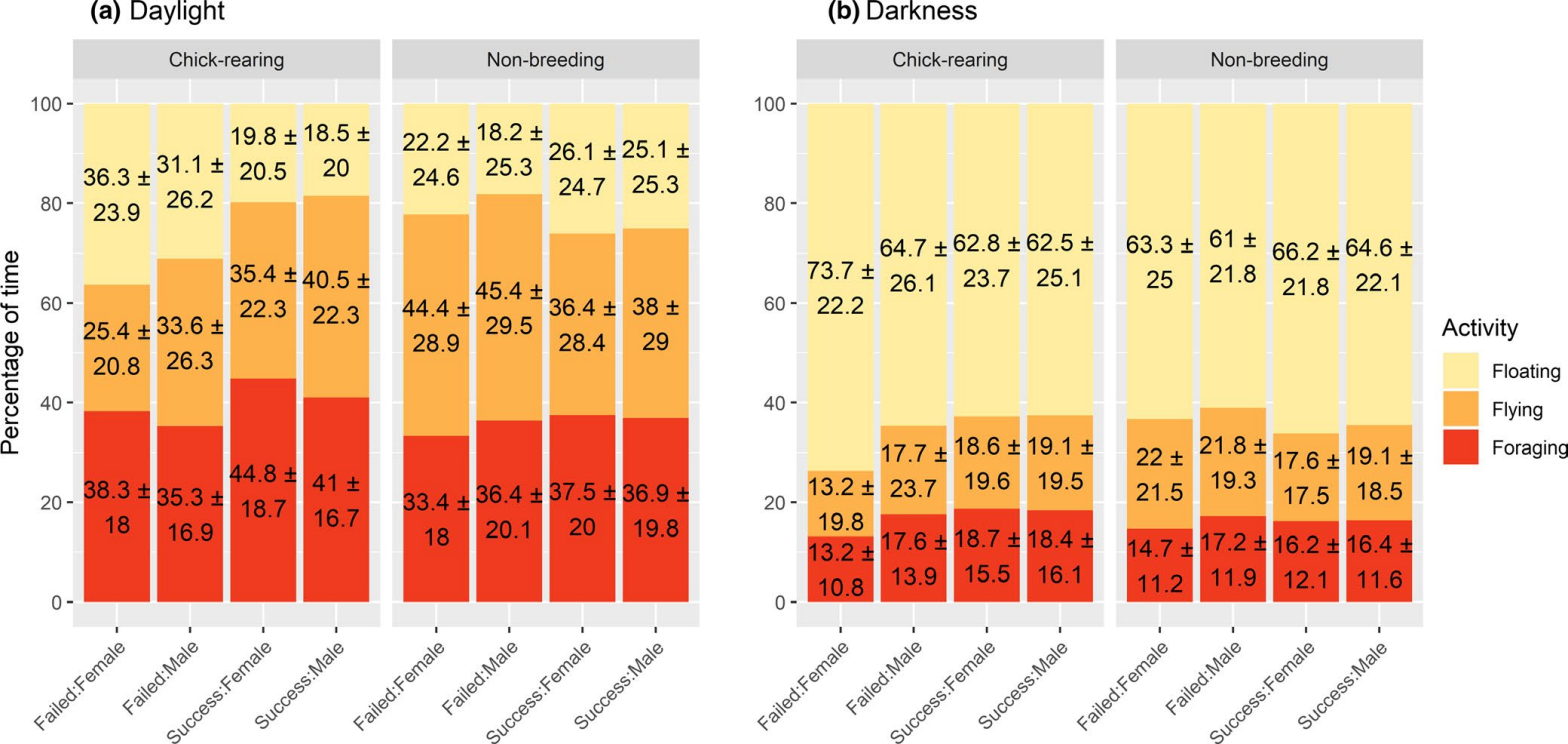
Mean ± *SD* percentage of time spent foraging, floating, and flying for (a) daylight and (b) darkness according breeding performance, sex, and annual cycle (chick‐rearing: 28/01/2012 to 25/02/2013; nonbreeding: 24/06/2013 to 21/07/2013)

Regarding the temporal dynamics of activity budgets (Figure 3, Table 2; full tables presented in Supplementary material), successful breeders spent as much time as failed breeders in foraging activities during daylight and darkness over the annual cycle (Figure 3), except during chick‐rearing, during which successful breeders increased their foraging effort (Figure 3a,d). Likewise, the two groups had similar dynamics in flying and floating activities at night with a decrease in flying and an increase in floating between the chick‐rearing and the nonbreeding period (Figures 3e,f and 4). Contrastingly, there were significant differences in the temporal dynamics of flying and floating activities during daylight during the chick‐rearing period (Figure 3b,c). Failed breeders rapidly reached their maximum floating activity during chick‐rearing, approximately 2 months after hatching, while successful breeders reached their maximum floating activity more than 2 months after failed breeders, during the nonbreeding season (Figure 3b,c). Flying activities were strongly and negatively correlated with floating activities, both during daylight and darkness and for both groups: When flying activities were maximal, floating activities were concurrently minimal and conversely, when floating activities were maximal, flying activities were concurrently minimal. Foraging seemed to be more independent.

**Table 2:** Summary statistics of the generalized additive mixed models for (a) nonlinear terms and (b) linear terms

Model	Smooth term	edf	ref.df	*F*	*p*‐value
(a)					
Foraging during daylight	ti(TimeSinceLaying):failed	9.35	10.42	8.82	<0.001
	ti(TimeSinceLaying):success	10.91	11.00	54.03	<0.001
	ti(MoonCycle)	1.53	3.00	1.7	0.029
Flying during daylight	ti(TimeSinceLaying):failed	10.54	10.95	54.81	<0.001
	ti(TimeSinceLaying):success	10.94	11.00	173.36	<0.001
	ti(MoonCycle)	2.97	3	9.76	<0.001
Floating during daylight	ti(TimeSinceLaying):failed	10.81	10.99	39.76	<0.001
	ti(TimeSinceLaying):success	10.97	11.00	137.26	<0.001
	ti(MoonCycle)	2.97	3.00	40.78	<0.001
Foraging at night	ti(TimeSinceLaying):failed	8.14	9.49	14.28	<0.001
	ti(TimeSinceLaying):success	10.63	10.97	37.54	<0.001
	ti(MoonCycle)	2.71	3.00	67.20	<0.001
Flying at night	ti(TimeSinceLaying):failed	9.90	10.72	8.08	<0.001
	ti(TimeSinceLaying):success	10.92	11.00	61.23	<0.001
	ti(MoonCycle)	2.86	3.00	231.02	<0.001
Floating at night	ti(TimeSinceLaying):failed	10.08	10.08	17.97	<0.001
	ti(TimeSinceLaying):success	10.94	11.00	97.41	<0.001
	ti(MoonCycle)	2.92	3.00	303.27	<0.001

	Model term	Estimate	*SE*	*t*‐value	*p*‐value
(b)					
Foraging daylight	(Intercept)	−0.66	0.05	−13.73	<0.001
	Statussuccess	0.16	0.05	2.99	0.003
Flying daylight	(Intercept)	−0.48	0.04	−11.80	<0.001
	Statussuccess	−0.14	0.05	−3.03	0.002
Floating daylight	(Intercept)	−1.20	0.06	−18.63	<0.001
	Statussuccess	0.00	0.07	−0.014	0.989
Foraging darkness	(Intercept)	−1.71	0.08	−21.57	<0.001
	Statussuccess	0.069	0.09	0.79	0.432
Flying darkness	(Intercept)	−1.49	0.05	−27.46	<0.001
	Statussuccess	−0.022	0.06	−0.36	0.717
Floating darkness	(Intercept)	0.62	0.07	9.50	<0.001
	Statussuccess	−0.028	0.07	−0.38	0.708

A comparison of activities between one moon cycle during chick‐rearing and one during the nonbreeding season confirmed that individuals were more active during daylight, spending less time floating on the water, and conversely, spending more time actively flying and foraging (Figure 4; Table S2). Individuals generally foraged significantly more during chick‐rearing than during the nonbreeding season (Figure 4; Table S2). Sex was not significant, except for flying activities, where successful males tended to fly more than successful females during daylight. There was also a significant interaction between sex and breeding status, with failed females foraging less and resting more than the three other groups, regardless of the annual cycle (Figure 4, Table S2). These results should be taken with caution due to the small sample size when splitting individuals by sex. Overall though, our results clearly showed greater differences between breeding states than sexes.

Based on activity data, the mean return date was similar between failed and successful breeders (9 September ± 16.2 days and 11 September ± 13.0 days, respectively; GLM; *F*_1,55_ = 0.76; *p* = 0.39) but males returned earlier than females (7 September ± 10.2 days and 16 September ± 15.3 days; GLM; *F*_1,54_ = 6.59; *p* = 0.01) and failed males returned even earlier (27 August ± 9 days; GLM; *F*_1,53_ = 5.69; *p* = 0.02).

### 3.3 Effect of the moon cycle

**Figure 5:**
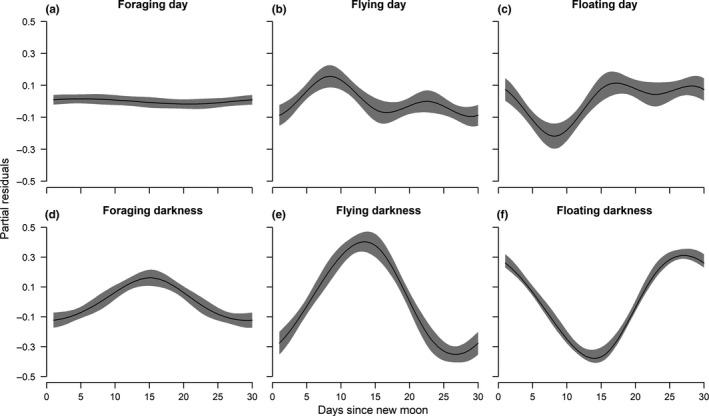
Effect of the moon cycle on the proportion of time spent foraging, flying, and floating on the water during daylight (upper panel) and darkness periods (lower panel), regardless of individual breeding performance. Day 1 corresponds to new moon while day 15 corresponds to full moon. Shaded areas represent 95% confidence intervals

The moon cycle had a significant effect on individual activity budgets during darkness, but also during daylight (Figure 5). During daylight, foraging effort was relatively constant over the moon cycle (Figure 5a) but flying activities increased around first and last quarters while floating concurrently decreased (Figure 5b,c). As expected, during darkness, the proportion of time spent flying and foraging increased around full moon while the proportion of time spent floating on the water concurrently decreased (Figure 5d–f).

## 4 DISCUSSION

Based on light and activity data obtained from geolocators, we examined the spatial distribution and temporal dynamics of activity budgets of failed and successfully breeding black‐browed albatrosses over the breeding and subsequent nonbreeding season.

### 4.1 Spatial distribution over the annual cycle

The 60 tracked black‐browed albatrosses remained on average 455–585 km away from their nesting colony during the study period, which is similar to the distance previously estimated with geolocators for six birds from the same colony (Grémillet et al., 2000). Males tended to remain closer to the colony than females but this result has to be taken cautiously due to measurement error of geolocators, which is ~200 km (Phillips et al., 2004).

The relatively limited range of the population all year‐round confirms that black‐browed albatrosses nesting in New Island can be considered as resident and do not undertake long‐distance dispersive migration, in great contrast to other populations (Desprez et al., 2018; Mackley et al., 2010; Phillips et al., 2005). The Patagonian shelf is well known as a highly productive marine area all year‐round (Acha, Mianzan, Guerrero, Favero, & Bava, 2004; Romero, Piola, Charo, & Garcia, 2006) and attracts a large number of seabird species, especially during winter (Berrow, Wood, & Prince, 2000; Croxall, Silk, Phillips, Afanasyev, & Briggs, 2005; Croxall & Wood, 2002; Guilford et al., 2009; Nicholls et al., 2002; Phillips et al., 2007; Phillips, Silk, Croxall, & Afanasyev, 2006), including some black‐browed albatrosses from the South Georgian population (Phillips et al., 2005). Thereby, black‐browed albatrosses nesting on New Island may benefit from excellent food resources year‐round and may not have to invest in costly long‐distance migration to find productive foraging areas and replenish their body reserves after reproduction. Moreover, we did not find any spatial segregation, neither by sex nor breeding status. This suggests that the Patagonian shelf is sufficiently productive to provide food for all individuals and that competition is not a limiting factor to food resource accessibility during the nonbreeding season, as it could be for other migrant albatross species (e.g., Clay et al., 2016; Phillips et al., 2005).

Despite an absence of directional migration, three individuals ventured toward the Chilean coast and South Georgia less than a month before returning to their home range. These trips indicate that studied individuals are still capable of long‐distant trips to reach different foraging grounds. These movements might be linked to specific nutritional requirements, conditioning subsequent breeding success (Desprez et al., 2018) but do not seem to be linked with previous breeding performance nor sex.

### 4.2 Temporal dynamics of activity budgets

As expected, resident black‐browed albatrosses decreased their general activity well before the end of the chick‐rearing period and did not face a peak of flying activity to reach and leave their nonbreeding ground, as other migratory procellariiform species usually do (e.g., Fayet et al., 2016; Gutowsky et al., 2014; Mackley et al., 2010; Péron et al., 2010; Ramos et al., 2018). Breeding status also strongly affected the temporal dynamics of activity budgets. Initially, both successful and failed breeders had similar activity budgets during incubation, when most failed birds had not yet lost their egg. The two groups reached maximal effort in flying at the same time during the brooding period. However, failed breeders reached their maximal peak of floating more rapidly than successful breeders. Moreover, contrarily to successful breeders, they did not spent more time foraging after the chick‐rearing period. Such differences in general activity budgets during the second part of the breeding season and the beginning of the nonbreeding season indicate that successful breeders worked harder during chick‐rearing: They spent more time flying between their foraging grounds and their colony to feed the chick. During chick‐rearing, they allocated more time to foraging both by day and night for 2 months, possibly to replenish their body reserves and prepare for molting (Catry, Poisbleau, Lecoq, & Phillips, 2013b). This further delayed their timing of minimal flying activity and maximal floating activities compared to failed breeders. Those results suggests that the response to breeding failure was an earlier change in time and energy allocation strategies: Successful breeders recovered from potential reproductive costs by allocating more time to foraging by day and night at the end of the chick‐rearing period and conversely delayed and reduced the duration of their resting period, when floating activity was maximum. On the contrary, failed breeders did not increase their foraging effort but rapidly decreased their general activity by spending more time floating on the water and less time flying and for a longer period, especially during daylight, which potentially allowed them to molt earlier and have more time to recover from those costs (Catry, Poisbleau, et al., 2013b; Ramos et al., 2018). These readjustments in activity budgets contrast with a recent study carried out on migrant black‐browed albatrosses nesting in Kerguelen (Southern Indian Ocean) that described a different effect of breeding failure on individual activity budgets (Desprez et al., 2018): Failed individuals increased their foraging effort, potentially because of a lower body condition. In our case, the main difference observed in the activity budgets between successful and failed breeders might not be linked to individual body condition, as failed individuals do not increase their foraging effort during the nonbreeding season. Foraging activities of failed females were even lower than other groups, suggesting that they did not need to feed more to compensate a potentially lower body condition. Consequently, residency may help accelerate buffering of reproductive costs, especially if environmental conditions are favorable (Ramos et al., 2018).

We also found that males, and failed males in particular, returned earlier to the colony. This is in line with patterns found in South Georgia and Kerguelen black‐browed albatrosses populations (Desprez et al., 2018; Phillips et al., 2005). However, we were unable to link return dates with subsequent breeding performance, as individual laying dates were unknown.

### 4.3 Daylight and moon cycle

As expected, regardless of individual breeding performance or sex, individuals were mainly diurnal. While the range of mean proportion of time spent flying was 25%–44% during daylight, it decreased to 13%–22% during darkness, with a general decrease from the chick‐rearing to the nonbreeding period. Although the birds did not migrate, their general proportion of time spent flying during the nonbreeding season was still higher than time spent floating on the water during daylight. This indicates that birds primarily move between different foraging patches during daylight. Additionally, full moon positively affected the general activity of birds at night, as they spent more time flying and foraging. These results are in accordance with other studies which showed a positive effect of full moon on procellariiform nocturnal activity (Dias et al., 2012; Hedd et al., 2001; Pinet et al., 2011; Yamamoto et al., 2008). Indeed, albatrosses, and especially black‐browed albatrosses, are thought to mainly rely on visual cues to catch their prey, and therefore, darkness may decrease their foraging efficiency, especially during new moon (Mackley et al., 2010; Phalan et al., 2007). While birds were more active at night during full moon, they did not correspondingly decrease their activity during daylight. This further suggests that foraging activities at night may be opportunistic. Full moon may attract an additional source of prey such as demersal fish and squids, which may become more easily accessible for black‐browed albatrosses, close to the surface.

Overall, our study highlights that although black‐browed albatrosses breeding in New Island do not undertake long‐distance migration, breeding performance still affects time and energy that individuals allocate to different activities, both during the breeding and the nonbreeding period. Successful breeders likely work harder during chick‐rearing, delaying, and shortening their resting phases compared to failed breeders. Nevertheless, these readjustments do not appear to lead individuals to segregate spatially during the nonbreeding season, outlining the high productivity, and thus, the high conservation value of the Patagonian shelf as a marine habitat (Grémillet et al., 2000).

## CONFLICT OF INTEREST

The authors declare no competing interests.

## AUTHOR CONTRIBUTIONS

PC and JPG conceived the study and conducted fieldwork. AH processed light data. AP processed activity data and conducted statistical analysis with the help of TC. AP wrote the manuscript with input from all co‐authors.

## Data Availability

Location data are deposited on the Seabird Tracking database handled by BirdLife International and activity data, on GitHub (https://github.com/auponchon/BBA-activity-data-2012-2013).
