# Supplementary material for: Effect of breeding performance on the distribution and activity budgets of a predominantly resident population of black‐browed albatrosses
Source: Ecol Evol. 2019 Jul 17;9(15):8702–13. doi: 10.1002/ece3.5416 (PMC6686306; doi:10.1002/ece3.5416)
Supplement: Supplementary file 1 [file ECE3-9-8702-s001.pdf]

Supporting Information

Effect of breeding performance on the distribution and activity budgets of a predominantly resident population of black-browed albatrosses

Aurore Ponchon , Thomas Cornulier, April Hedd, José Pedro Granadeiro, Paulo Catry

Figure S1: Representativeness of the number of tracked individuals (sample size) for (a) successful breeders, (b) failed breeders, (c) males and (d) females obtained from a bootstrap analysis with 100 iterations. Circles indicate the average proportion (inclusion) of out-of-sample positions located within the 90% UDs estimated from sampled positions and grey shadow indicates the standard deviation.

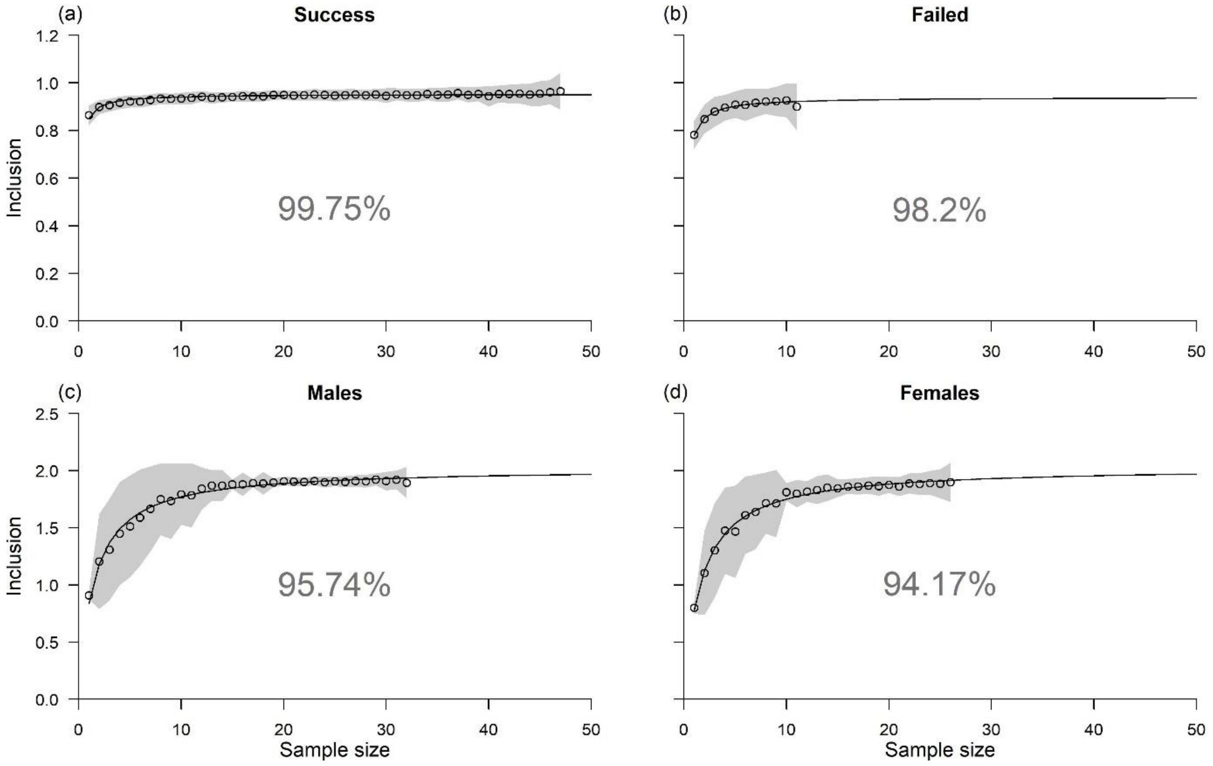

Figure S2: Weekly average ± SD of the distance to the colony for successful female (dashed blue), successful male (solid blue), failed female (dashed red) and failed male (solid red) black-browed albatrosses nesting in New Island (Falklands' archipelago). Data are missing around equinox, as locations are unreliable for this period.

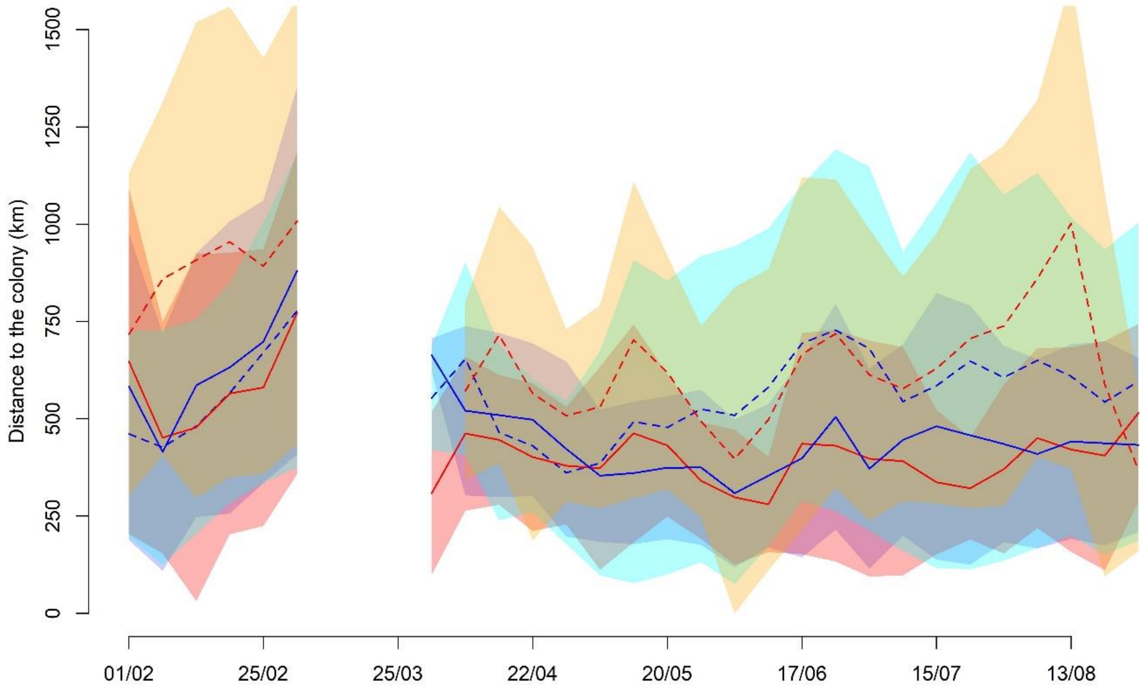

Table S1: Laying date, hatching date and/or date of failure for all individuals included in the analysis

Individual	Sex	Status	Laying date	Hatching date	Date of failure
**B30P**	male	failed	07/10/2012	-	01/01/2013
**R49C**	male	failed	07/10/2012	-	20/01/2013
**R53A**	male	failed	11/10/2012	-	26/12/2012
**R61A**	male	failed	16/10/2012	-	20/12/2012
**R72A**	female	failed	10/10/2012	-	09/01/2013
**W05K**	male	failed	07/10/2012	-	05/12/2012
**W29E**	female	failed	08/10/2012	-	27/10/2012
**W71K**	male	failed	08/10/2012	-	23/12/2012
**W77K**	female	failed	09/10/2012	-	08/01/2013
**W91K**	female	failed	07/10/2012	-	04/12/2012
**W52K**	female	failed	10/10/2012	17/12/2012	23/12/2012
**W65E**	female	failed	07/10/2012	12/12/2012	27/02/2013
**B04P**	female	success	15/10/2012	24/12/2012	-
**B14P**	female	success	10/10/2012	18/12/2012	-
**B16P**	male	success	09/10/2012	17/12/2012	-
**B18P**	female	success	10/10/2012	18/12/2012	-
**B19P**	male	success	16/10/2012	24/12/2012	-
**B29P**	female	success	10/10/2012	18/12/2012	-
**B52P**	female	success	09/10/2012	15/12/2012	-
**B65P**	female	success	12/10/2012	20/12/2012	-
**B68P**	female	success	11/10/2012	18/12/2012	-
**R01C**	male	success	12/10/2012	19/12/2012	-
**R02A**	male	success	11/10/2012	19/12/2012	-
**R08A**	female	success	10/10/2012	20/12/2012	-
**R14A**	male	success	14/10/2012	21/12/2012	-
**R23A**	male	success	11/10/2012	20/12/2012	-
**R30A**	male	success	09/10/2012	17/12/2012	-
**R37A**	male	success	12/10/2012	20/12/2012	-
**R39C**	male	success	11/10/2012	19/12/2012	-
**R40C**	female	success	13/10/2012	21/12/2012	-
**R44C**	male	success	09/10/2012	13/12/2012	-
**R46C**	male	success	10/10/2012	19/12/2012	-
**R54A**	female	success	10/10/2012	18/12/2012	-
**R59C**	male	success	14/10/2012	22/12/2012	-
**R60A**	male	success	09/10/2012	15/12/2012	-
**R62A**	female	success	13/10/2012	22/12/2012	-
**R62C**	female	success	16/10/2012	24/12/2012	-
**R63C**	male	success	09/10/2012	12/12/2012	-

**R64C**	female	success	12/10/2012	20/12/2012	-
**R85C**	male	success	09/10/2012	13/12/2012	-
**R91A**	male	success	10/10/2012	18/12/2012	-
**R94A**	male	success	12/10/2012	19/12/2012	-
**W02E**	female	success	13/10/2012	20/12/2012	-
**W04E**	male	success	13/10/2012	21/12/2012	-
**W12K**	male	success	10/10/2012	19/12/2012	-
**W14K**	female	success	15/10/2012	24/12/2012	-
**W20E**	male	success	10/10/2012	17/12/2012	-
**W25K**	female	success	11/10/2012	20/12/2012	-
**W26K**	male	success	11/10/2012	20/12/2012	-
**W30K**	male	success	09/10/2012	12/12/2012	-
**W46E**	male	success	12/10/2012	20/12/2012	-
**W57E**	female	success	09/10/2012	17/12/2012	-
**W58K**	male	success	16/10/2012	22/12/2012	-
**W60E**	male	success	09/10/2012	15/12/2012	-
**W72K**	female	success	14/10/2012	22/12/2012	-
**W85K**	male	success	15/10/2012	24/12/2012	-
**W92K**	female	success	13/10/2012	23/12/2012	-
**W97K**	male	success	11/10/2012	18/12/2012	-
**W99E**	female	success	12/10/2012	19/12/2012	-
**Y79L**	female	success	12/10/2012	20/12/2012	-

Table S2: Summary statistics of the generalized additive mixed models for the non-linear of the GAMM

	Smooth term	edf	ref.df	F	p-value
**Proportion of time spent foraging during daylight**	ti(TimeSinceLaying):Statusfailed	9.35	10.42	8.82	< 0.001
	ti(TimeSinceLaying):Statussuccess	10.91	11.00	54.03	< 0.001
	ti(MoonCycle)	1.53	3.00	1.70	0.029
	ti(TimeSinceLaying):IDY79L	3.46	3.84	6.36	< 0.001
	ti(TimeSinceLaying):IDW99E	1.98	2.43	1.85	0.11
	ti(TimeSinceLaying):IDW97K	2.35	2.85	4.81	0.002
	ti(TimeSinceLaying):IDW92K	3.47	3.84	6.14	< 0.001
	ti(TimeSinceLaying):IDW91K	1.00	1.00	14.29	< 0.001
	ti(TimeSinceLaying):IDW85K	1.00	1.00	1.14	0.29
	ti(TimeSinceLaying):IDW77K	2.76	3.27	1.09	0.41
	ti(TimeSinceLaying):IDW72K	2.49	3.00	2.85	0.036
	ti(TimeSinceLaying):IDW71K	1.92	2.35	2.69	0.058
	ti(TimeSinceLaying):IDW65E	3.35	3.76	3.08	0.022
	ti(TimeSinceLaying):IDW60E	3.84	3.98	5.68	< 0.001
	ti(TimeSinceLaying):IDW58K	1.18	1.33	0.66	0.36
	ti(TimeSinceLaying):IDW57E	3.87	3.99	5.24	< 0.001
	ti(TimeSinceLaying):IDW52K	1.00	1.00	2.13	0.14
	ti(TimeSinceLaying):IDW46E	2.56	3.08	5.90	< 0.001
	ti(TimeSinceLaying):IDW30K	1.00	1.00	3.74	0.053
	ti(TimeSinceLaying):IDW29E	2.84	3.35	1.72	0.14
	ti(TimeSinceLaying):IDW26K	1.92	2.36	0.63	0.69
	ti(TimeSinceLaying):IDW25K	3.47	3.84	0.69	0.53
	ti(TimeSinceLaying):IDW20E	1.43	1.73	0.58	0.63
	ti(TimeSinceLaying):IDW14K	1.00	1.00	0.58	0.44
	ti(TimeSinceLaying):IDW12K	1.17	1.32	0.39	0.50
	ti(TimeSinceLaying):IDW05K	3.88	3.99	5.61	< 0.001
	ti(TimeSinceLaying):IDW04E	1.00	1.00	0.19	0.67
	ti(TimeSinceLaying):IDW02E	3.71	3.95	9.66	< 0.001
	ti(TimeSinceLaying):IDR94A	1.00	1.00	0.07	0.79
	ti(TimeSinceLaying):IDR91A	1.00	1.00	1.70	0.19
	ti(TimeSinceLaying):IDR85C	1.00	1.00	1.51	0.22
	ti(TimeSinceLaying):IDR72A	1.00	1.00	0.25	0.62
	ti(TimeSinceLaying):IDR64C	3.56	3.89	5.80	< 0.001
	ti(TimeSinceLaying):IDR63C	1.77	2.18	1.21	0.31
	ti(TimeSinceLaying):IDR62C	3.01	3.51	1.86	0.093
	ti(TimeSinceLaying):IDR62A	3.87	3.99	6.69	< 0.001
	ti(TimeSinceLaying):IDR61A	1.00	1.00	4.48	0.034
	ti(TimeSinceLaying):IDR60A	0.00	0.00	0.00	1.00
	ti(TimeSinceLaying):IDR59C	2.21	2.70	1.59	0.13

	ti(TimeSinceLaying):IDR54A	3.73	3.95	9.84	< 0.001
	ti(TimeSinceLaying):IDR53A	2.29	2.79	1.04	0.51
	ti(TimeSinceLaying):IDR49C	3.74	3.96	2.58	0.026
	ti(TimeSinceLaying):IDR46C	2.00	2.46	2.11	0.13
	ti(TimeSinceLaying):IDR44C	3.48	3.84	2.45	0.034
	ti(TimeSinceLaying):IDR40C	1.00	1.00	0.50	0.48
	ti(TimeSinceLaying):IDR39C	1.30	1.54	1.17	0.21
	ti(TimeSinceLaying):IDR37A	3.43	3.81	1.23	0.20
	ti(TimeSinceLaying):IDR30A	1.37	1.65	1.04	0.44
	ti(TimeSinceLaying):IDR23A	3.24	3.69	4.54	0.002
	ti(TimeSinceLaying):IDR14A	1.00	1.00	1.60	0.21
	ti(TimeSinceLaying):IDR08A	1.00	1.00	0.13	0.72
	ti(TimeSinceLaying):IDR02A	3.10	3.58	1.94	0.16
	ti(TimeSinceLaying):IDR01C	1.62	1.99	2.82	0.068
	ti(TimeSinceLaying):IDB68P	1.00	1.00	5.10	0.024
	ti(TimeSinceLaying):IDB65P	1.00	1.01	0.01	0.92
	ti(TimeSinceLaying):IDB52P	3.83	3.98	4.48	0.001
	ti(TimeSinceLaying):IDB30P	0.42	0.72	1.02	0.39
	ti(TimeSinceLaying):IDB29P	1.02	1.04	0.56	0.47
	ti(TimeSinceLaying):IDB19P	3.64	3.92	1.60	0.14
	ti(TimeSinceLaying):IDB18P	1.00	1.00	0.06	0.80
	ti(TimeSinceLaying):IDB16P	1.00	1.00	0.63	0.43
	ti(TimeSinceLaying):IDB14P	3.76	3.96	3.34	0.007
	ti(TimeSinceLaying):IDB04P	3.85	3.99	4.32	0.002
	s(ID)	53.19	58.00	10.70	< 0.001
**Proportion of time spent flying during daylight**	ti(TimeSinceLaying):Statusfailed	10.54	10.95	54.81	< 0.001
	ti(TimeSinceLaying):Statussuccess	10.94	11.00	173.36	< 0.001
	ti(MoonCycle)	2.97	3.00	26.30	< 0.001
	ti(TimeSinceLaying):IDY79L	3.93	4.00	9.76	< 0.001
	ti(TimeSinceLaying):IDW99E	1.00	1.00	10.82	0.001
	ti(TimeSinceLaying):IDW97K	2.97	3.47	3.72	0.013
	ti(TimeSinceLaying):IDW92K	2.85	3.36	4.00	0.006
	ti(TimeSinceLaying):IDW91K	1.50	1.83	1.45	0.33
	ti(TimeSinceLaying):IDW85K	1.48	1.80	0.37	0.59
	ti(TimeSinceLaying):IDW77K	1.00	1.00	0.79	0.37
	ti(TimeSinceLaying):IDW72K	2.95	3.46	3.22	0.013
	ti(TimeSinceLaying):IDW71K	1.76	2.17	6.03	0.002
	ti(TimeSinceLaying):IDW65E	0.00	0.00	0.12	0.99
	ti(TimeSinceLaying):IDW60E	2.02	2.48	1.56	0.27
	ti(TimeSinceLaying):IDW58K	1.00	1.00	0.25	0.62
	ti(TimeSinceLaying):IDW57E	2.27	2.76	2.54	0.11
	ti(TimeSinceLaying):IDW52K	2.92	3.42	1.38	0.19
	ti(TimeSinceLaying):IDW46E	1.00	1.00	2.32	0.13
	ti(TimeSinceLaying):IDW30K	1.69	2.08	3.06	0.041

ti(TimeSinceLaying):IDW29E	3.71	3.95	4.65	0.003
ti(TimeSinceLaying):IDW26K	3.66	3.93	1.84	0.10
ti(TimeSinceLaying):IDW25K	1.00	1.00	0.28	0.60
ti(TimeSinceLaying):IDW20E	1.02	1.48	2.26	0.069
ti(TimeSinceLaying):IDW14K	1.00	1.00	0.04	0.84
ti(TimeSinceLaying):IDW12K	3.59	3.90	2.37	0.031
ti(TimeSinceLaying):IDW05K	3.87	3.99	16.45	< 0.001
ti(TimeSinceLaying):IDW04E	1.00	1.00	0.60	0.44
ti(TimeSinceLaying):IDW02E	3.52	3.87	3.44	0.006
ti(TimeSinceLaying):IDR94A	3.56	3.89	3.24	0.007
ti(TimeSinceLaying):IDR91A	1.00	1.00	4.80	0.028
ti(TimeSinceLaying):IDR85C	1.00	1.00	1.79	0.18
ti(TimeSinceLaying):IDR72A	1.00	1.00	0.10	0.75
ti(TimeSinceLaying):IDR64C	3.91	3.99	8.58	< 0.001
ti(TimeSinceLaying):IDR63C	1.39	1.67	1.14	0.42
ti(TimeSinceLaying):IDR62C	1.00	1.00	0.11	0.74
ti(TimeSinceLaying):IDR62A	1.00	1.00	3.12	0.077
ti(TimeSinceLaying):IDR61A	3.06	3.54	4.39	0.002
ti(TimeSinceLaying):IDR60A	2.70	3.22	1.34	0.28
ti(TimeSinceLaying):IDR59C	3.27	3.72	4.14	0.003
ti(TimeSinceLaying):IDR54A	3.21	3.67	5.59	< 0.001
ti(TimeSinceLaying):IDR53A	1.59	1.95	0.43	0.64
ti(TimeSinceLaying):IDR49C	1.88	2.31	0.86	0.36
ti(TimeSinceLaying):IDR46C	3.54	3.88	2.56	0.037
ti(TimeSinceLaying):IDR44C	3.93	4.00	14.77	< 0.001
ti(TimeSinceLaying):IDR40C	3.90	3.99	7.17	< 0.001
ti(TimeSinceLaying):IDR39C	1.24	1.44	0.78	0.31
ti(TimeSinceLaying):IDR37A	1.00	1.00	1.34	0.25
ti(TimeSinceLaying):IDR30A	1.00	1.00	0.65	0.42
ti(TimeSinceLaying):IDR23A	2.93	3.44	3.03	0.027
ti(TimeSinceLaying):IDR14A	3.29	3.73	2.48	0.057
ti(TimeSinceLaying):IDR08A	3.84	3.98	9.96	< 0.001
ti(TimeSinceLaying):IDR02A	2.25	2.75	1.41	0.21
ti(TimeSinceLaying):IDR01C	1.00	1.00	8.49	0.004
ti(TimeSinceLaying):IDB68P	1.68	2.07	1.68	0.19
ti(TimeSinceLaying):IDB65P	1.00	1.00	6.53	0.011
ti(TimeSinceLaying):IDB52P	3.95	4.00	6.41	< 0.001
ti(TimeSinceLaying):IDB30P	2.72	3.22	0.83	0.44
ti(TimeSinceLaying):IDB29P	1.88	2.32	1.29	0.23
ti(TimeSinceLaying):IDB19P	3.63	3.92	1.82	0.098
ti(TimeSinceLaying):IDB18P	1.00	1.00	3.98	0.046
ti(TimeSinceLaying):IDB16P	1.00	1.00	2.09	0.15
ti(TimeSinceLaying):IDB14P	3.95	4.00	7.66	< 0.001
ti(TimeSinceLaying):IDB04P	1.00	1.00	4.60	0.032
s(ID)	44.22	58.00	3.60	< 0.001

**Proportion of time spent floating during daylight**	ti(TimeSinceLaying):Statusfailed	10.81	10.99	39.76	< 0.001
	ti(TimeSinceLaying):Statussuccess	10.97	11.00	137.26	< 0.001
	ti(MoonCycle)	2.97	3.00	40.78	< 0.001
	ti(TimeSinceLaying):IDY79L	3.90	3.99	16.90	< 0.001
	ti(TimeSinceLaying):IDW99E	2.10	2.57	1.62	0.16
	ti(TimeSinceLaying):IDW97K	2.96	3.46	7.55	< 0.001
	ti(TimeSinceLaying):IDW92K	2.65	3.16	0.55	0.59
	ti(TimeSinceLaying):IDW91K	3.76	3.96	4.16	0.004
	ti(TimeSinceLaying):IDW85K	2.23	2.71	3.18	0.024
	ti(TimeSinceLaying):IDW77K	3.60	3.90	6.10	< 0.001
	ti(TimeSinceLaying):IDW72K	1.00	1.01	0.88	0.35
	ti(TimeSinceLaying):IDW71K	3.13	3.59	5.40	< 0.001
	ti(TimeSinceLaying):IDW65E	3.79	3.97	9.70	< 0.001
	ti(TimeSinceLaying):IDW60E	3.08	3.55	1.58	0.25
	ti(TimeSinceLaying):IDW58K	1.29	1.52	1.85	0.11
	ti(TimeSinceLaying):IDW57E	3.91	3.99	5.90	< 0.001
	ti(TimeSinceLaying):IDW52K	1.00	1.00	0.26	0.61
	ti(TimeSinceLaying):IDW46E	3.41	3.80	3.43	0.006
	ti(TimeSinceLaying):IDW30K	3.01	3.50	1.51	0.14
	ti(TimeSinceLaying):IDW29E	1.00	1.00	1.54	0.22
	ti(TimeSinceLaying):IDW26K	3.83	3.98	3.88	0.003
	ti(TimeSinceLaying):IDW25K	1.54	1.88	3.44	0.060
	ti(TimeSinceLaying):IDW20E	1.74	2.15	2.60	0.070
	ti(TimeSinceLaying):IDW14K	1.00	1.00	3.79	0.051
	ti(TimeSinceLaying):IDW12K	3.39	3.78	3.59	0.005
	ti(TimeSinceLaying):IDW05K	4.00	4.00	21.54	< 0.001
	ti(TimeSinceLaying):IDW04E	1.00	1.00	1.38	0.24
	ti(TimeSinceLaying):IDW02E	1.85	2.27	1.22	0.26
	ti(TimeSinceLaying):IDR94A	3.39	3.79	4.71	0.001
	ti(TimeSinceLaying):IDR91A	1.61	1.97	0.63	0.52
	ti(TimeSinceLaying):IDR85C	3.45	3.82	1.98	0.18
	ti(TimeSinceLaying):IDR72A	3.44	3.82	2.75	0.018
	ti(TimeSinceLaying):IDR64C	3.90	3.99	11.16	< 0.001
	ti(TimeSinceLaying):IDR63C	1.00	1.00	0.00	0.95
	ti(TimeSinceLaying):IDR62C	2.82	3.32	2.78	0.028
	ti(TimeSinceLaying):IDR62A	3.78	3.97	3.97	0.002
	ti(TimeSinceLaying):IDR61A	2.24	2.73	3.56	0.034
	ti(TimeSinceLaying):IDR60A	2.09	2.55	0.90	0.33
	ti(TimeSinceLaying):IDR59C	3.74	3.96	7.70	< 0.001
	ti(TimeSinceLaying):IDR54A	3.24	3.69	1.64	0.15
	ti(TimeSinceLaying):IDR53A	1.70	2.10	2.62	0.077
	ti(TimeSinceLaying):IDR49C	3.00	3.00	8.50	< 0.001
	ti(TimeSinceLaying):IDR46C	1.10	1.18	1.05	0.35
	ti(TimeSinceLaying):IDR44C	2.98	3.00	32.80	< 0.001
	ti(TimeSinceLaying):IDR40C	3.64	3.92	4.93	< 0.001

	ti(TimeSinceLaying):IDR39C	1.00	1.00	1.91	0.17
	ti(TimeSinceLaying):IDR37A	1.00	1.00	0.86	0.35
	ti(TimeSinceLaying):IDR30A	1.00	1.00	1.99	0.16
	ti(TimeSinceLaying):IDR23A	2.28	2.75	6.39	< 0.001
	ti(TimeSinceLaying):IDR14A	3.66	3.93	2.42	0.043
	ti(TimeSinceLaying):IDR08A	3.66	3.93	10.03	< 0.001
	ti(TimeSinceLaying):IDR02A	3.29	3.73	4.18	0.004
	ti(TimeSinceLaying):IDR01C	2.65	3.16	2.57	0.059
	ti(TimeSinceLaying):IDB68P	1.55	1.89	2.59	0.054
	ti(TimeSinceLaying):IDB65P	2.55	3.06	2.90	0.034
	ti(TimeSinceLaying):IDB52P	3.92	4.00	12.41	< 0.001
	ti(TimeSinceLaying):IDB30P	1.00	1.01	5.51	0.019
	ti(TimeSinceLaying):IDB29P	1.01	1.01	0.03	0.86
	ti(TimeSinceLaying):IDB19P	1.00	1.00	0.78	0.38
	ti(TimeSinceLaying):IDB18P	1.00	1.00	0.02	0.88
	ti(TimeSinceLaying):IDB16P	1.00	1.00	0.65	0.42
	ti(TimeSinceLaying):IDB14P	3.94	4.00	13.18	< 0.001
	ti(TimeSinceLaying):IDB04P	1.00	1.00	1.57	0.21
	s(ID)	50.65	58.00	6.36	< 0.001
**Proportion of time spent foraging during darkness**	ti(TimeSinceLaying):Statusfailed	8.14	9.49	14.28	< 0.001
	ti(TimeSinceLaying):Statussuccess	10.63	10.97	37.54	< 0.001
	ti(MoonCycle)	2.71	3.00	67.20	< 0.001
	ti(TimeSinceLaying):IDY79L	3.28	3.72	6.09	< 0.001
	ti(TimeSinceLaying):IDW99E	3.65	3.93	3.08	0.032
	ti(TimeSinceLaying):IDW97K	1.30	1.54	3.92	0.022
	ti(TimeSinceLaying):IDW92K	1.00	1.00	0.29	0.59
	ti(TimeSinceLaying):IDW91K	3.13	3.60	1.55	0.13
	ti(TimeSinceLaying):IDW85K	1.38	1.65	0.81	0.55
	ti(TimeSinceLaying):IDW77K	2.50	3.01	3.63	0.012
	ti(TimeSinceLaying):IDW72K	3.73	3.96	1.89	0.077
	ti(TimeSinceLaying):IDW71K	1.00	1.00	5.90	0.015
	ti(TimeSinceLaying):IDW65E	3.09	3.57	6.67	< 0.001
	ti(TimeSinceLaying):IDW60E	1.00	1.00	0.33	0.56
	ti(TimeSinceLaying):IDW58K	1.00	1.00	0.47	0.50
	ti(TimeSinceLaying):IDW57E	1.00	1.00	0.55	0.46
	ti(TimeSinceLaying):IDW52K	1.00	1.00	1.38	0.24
	ti(TimeSinceLaying):IDW46E	3.60	3.90	1.80	0.14
	ti(TimeSinceLaying):IDW30K	1.77	2.18	1.05	0.34
	ti(TimeSinceLaying):IDW29E	2.11	2.57	5.07	0.003
	ti(TimeSinceLaying):IDW26K	3.37	3.78	0.88	0.35
	ti(TimeSinceLaying):IDW25K	2.24	2.73	2.17	0.066
	ti(TimeSinceLaying):IDW20E	3.74	3.96	3.80	0.006
	ti(TimeSinceLaying):IDW14K	2.13	2.60	1.90	0.12
	ti(TimeSinceLaying):IDW12K	3.21	3.67	2.66	0.025
	ti(TimeSinceLaying):IDW05K	3.07	3.53	2.84	0.022

	ti(TimeSinceLaying):IDW04E	3.78	3.97	1.72	0.13
	ti(TimeSinceLaying):IDW02E	1.09	1.18	2.05	0.17
	ti(TimeSinceLaying):IDR94A	3.64	3.92	5.94	< 0.001
	ti(TimeSinceLaying):IDR91A	3.42	3.81	1.22	0.40
	ti(TimeSinceLaying):IDR85C	3.23	3.68	3.46	0.010
	ti(TimeSinceLaying):IDR72A	1.78	2.29	7.28	0.001
	ti(TimeSinceLaying):IDR64C	1.00	1.00	1.28	0.26
	ti(TimeSinceLaying):IDR63C	1.01	1.02	1.27	0.25
	ti(TimeSinceLaying):IDR62C	1.82	2.24	1.19	0.30
	ti(TimeSinceLaying):IDR62A	1.97	2.43	3.09	0.038
	ti(TimeSinceLaying):IDR61A	1.63	2.00	2.14	0.11
	ti(TimeSinceLaying):IDR60A	0.37	0.65	0.86	0.46
	ti(TimeSinceLaying):IDR59C	3.49	3.85	5.72	< 0.001
	ti(TimeSinceLaying):IDR54A	3.89	3.99	4.85	0.001
	ti(TimeSinceLaying):IDR53A	1.00	1.01	6.24	0.012
	ti(TimeSinceLaying):IDR49C	1.00	1.00	13.26	< 0.001
	ti(TimeSinceLaying):IDR46C	1.00	1.00	0.23	0.63
	ti(TimeSinceLaying):IDR44C	1.36	1.64	4.20	0.047
	ti(TimeSinceLaying):IDR40C	3.32	3.75	3.52	0.007
	ti(TimeSinceLaying):IDR39C	3.47	3.84	1.86	0.071
	ti(TimeSinceLaying):IDR37A	1.00	1.00	0.70	0.40
	ti(TimeSinceLaying):IDR30A	1.00	1.01	1.20	0.27
	ti(TimeSinceLaying):IDR23A	3.65	3.92	7.21	< 0.001
	ti(TimeSinceLaying):IDR14A	2.83	3.34	1.91	0.16
	ti(TimeSinceLaying):IDR08A	3.76	3.96	6.27	< 0.001
	ti(TimeSinceLaying):IDR02A	2.33	2.83	2.44	0.10
	ti(TimeSinceLaying):IDR01C	2.14	2.61	3.08	0.029
	ti(TimeSinceLaying):IDB68P	1.00	1.00	0.12	0.73
	ti(TimeSinceLaying):IDB65P	3.37	3.78	6.02	< 0.001
	ti(TimeSinceLaying):IDB52P	3.94	4.00	8.01	< 0.001
	ti(TimeSinceLaying):IDB30P	3.91	3.99	5.76	< 0.001
	ti(TimeSinceLaying):IDB29P	3.51	3.86	4.59	0.003
	ti(TimeSinceLaying):IDB19P	3.10	3.59	2.51	0.053
	ti(TimeSinceLaying):IDB18P	3.66	3.93	3.99	0.006
	ti(TimeSinceLaying):IDB16P	3.73	3.96	5.80	< 0.001
	ti(TimeSinceLaying):IDB14P	3.61	3.91	2.19	0.082
	ti(TimeSinceLaying):IDB04P	1.00	1.00	9.25	0.002
	s(ID)	55.50	58.00	18.08	< 0.001
**Proportion of time spent flying during**	ti(TimeSinceLaying):Statusfailed	9.90	10.72	8.10	< 0.001
	ti(TimeSinceLaying):Statussuccess	10.92	11.00	61.23	< 0.001
	ti(MoonCycle)	2.86	3.00	231.02	< 0.001
	ti(TimeSinceLaying):IDY79L	3.21	3.66	4.46	0.002
	ti(TimeSinceLaying):IDW99E	1.70	2.09	0.50	0.57
	ti(TimeSinceLaying):IDW97K	2.28	2.76	4.62	0.004
	ti(TimeSinceLaying):IDW92K	1.00	1.00	4.28	0.039

ti(TimeSinceLaying):IDW91K	3.80	3.97	2.73	0.029
ti(TimeSinceLaying):IDW85K	3.87	3.99	3.33	0.011
ti(TimeSinceLaying):IDW77K	1.89	2.32	2.41	0.091
ti(TimeSinceLaying):IDW72K	3.42	3.81	3.17	0.009
ti(TimeSinceLaying):IDW71K	1.00	1.00	0.22	0.64
ti(TimeSinceLaying):IDW65E	0.00	0.00	0.01	1.00
ti(TimeSinceLaying):IDW60E	1.00	1.00	0.44	0.51
ti(TimeSinceLaying):IDW58K	2.87	3.39	6.24	< 0.001
ti(TimeSinceLaying):IDW57E	2.62	3.13	3.38	0.015
ti(TimeSinceLaying):IDW52K	1.00	1.00	1.55	0.21
ti(TimeSinceLaying):IDW46E	1.00	1.00	0.64	0.42
ti(TimeSinceLaying):IDW30K	2.88	3.38	1.13	0.42
ti(TimeSinceLaying):IDW29E	2.08	2.52	3.53	0.018
ti(TimeSinceLaying):IDW26K	2.78	3.29	3.04	0.020
ti(TimeSinceLaying):IDW25K	3.65	3.93	2.89	0.026
ti(TimeSinceLaying):IDW20E	1.95	2.39	2.83	0.047
ti(TimeSinceLaying):IDW14K	1.00	1.00	0.92	0.34
ti(TimeSinceLaying):IDW12K	1.00	1.00	0.52	0.47
ti(TimeSinceLaying):IDW05K	3.66	3.92	4.03	0.013
ti(TimeSinceLaying):IDW04E	3.33	3.75	3.84	0.011
ti(TimeSinceLaying):IDW02E	1.00	1.00	0.06	0.81
ti(TimeSinceLaying):IDR94A	1.00	1.00	0.02	0.89
ti(TimeSinceLaying):IDR91A	1.71	2.22	1.23	0.26
ti(TimeSinceLaying):IDR85C	3.87	3.99	4.47	0.001
ti(TimeSinceLaying):IDR72A	1.64	2.00	1.23	0.29
ti(TimeSinceLaying):IDR64C	1.00	1.00	4.88	0.027
ti(TimeSinceLaying):IDR63C	1.98	2.42	0.96	0.31
ti(TimeSinceLaying):IDR62C	3.75	3.96	2.80	0.027
ti(TimeSinceLaying):IDR62A	1.00	1.00	0.49	0.49
ti(TimeSinceLaying):IDR61A	3.25	3.67	5.54	< 0.001
ti(TimeSinceLaying):IDR60A	2.67	3.18	3.55	0.013
ti(TimeSinceLaying):IDR59C	2.58	3.09	1.15	0.293
ti(TimeSinceLaying):IDR54A	1.21	1.38	6.44	0.004
ti(TimeSinceLaying):IDR53A	3.22	3.66	1.15	0.45
ti(TimeSinceLaying):IDR49C	3.79	3.97	2.41	0.046
ti(TimeSinceLaying):IDR46C	1.86	2.29	1.16	0.26
ti(TimeSinceLaying):IDR44C	3.60	3.90	2.35	0.044
ti(TimeSinceLaying):IDR40C	2.09	2.56	1.95	0.15
ti(TimeSinceLaying):IDR39C	1.94	2.38	3.15	0.039
ti(TimeSinceLaying):IDR37A	1.00	1.00	5.90	0.015
ti(TimeSinceLaying):IDR30A	3.87	3.99	3.40	0.010
ti(TimeSinceLaying):IDR23A	3.52	3.87	5.78	< 0.001
ti(TimeSinceLaying):IDR14A	2.89	3.40	1.81	0.18
ti(TimeSinceLaying):IDR08A	2.22	2.71	3.45	0.020
ti(TimeSinceLaying):IDR02A	1.00	1.00	0.70	0.40

	ti(TimeSinceLaying):IDR01C	1.00	1.00	2.04	0.15
	ti(TimeSinceLaying):IDB68P	3.25	3.69	1.57	0.12
	ti(TimeSinceLaying):IDB65P	3.85	3.98	2.62	0.041
	ti(TimeSinceLaying):IDB52P	1.12	1.22	0.11	0.74
	ti(TimeSinceLaying):IDB30P	3.48	3.84	1.65	0.30
	ti(TimeSinceLaying):IDB29P	3.82	3.98	2.02	0.086
	ti(TimeSinceLaying):IDB19P	1.00	1.01	2.17	0.14
	ti(TimeSinceLaying):IDB18P	1.00	1.00	1.39	0.24
	ti(TimeSinceLaying):IDB16P	1.86	2.29	1.71	0.17
	ti(TimeSinceLaying):IDB14P	1.06	1.12	0.15	0.69
	ti(TimeSinceLaying):IDB04P	1.00	1.00	0.04	0.84
	s(ID)	47.48	58.00	4.69	< 0.001
**Proportion of time spent** floating during darkness	ti(TimeSinceLaying):Statusfailed	10.08	10.80	17.97	< 0.001
	ti(TimeSinceLaying):Statussuccess	10.94	11.00	97.41	< 0.001
	ti(MoonCycle)	2.92	3.00	303.27	< 0.001
	ti(TimeSinceLaying):IDY79L	3.30	3.74	5.06	< 0.001
	ti(TimeSinceLaying):IDW99E	2.42	2.92	6.89	< 0.001
	ti(TimeSinceLaying):IDW97K	2.76	3.28	6.56	< 0.001
	ti(TimeSinceLaying):IDW92K	1.00	1.00	0.12	0.73
	ti(TimeSinceLaying):IDW91K	3.87	3.99	7.71	< 0.001
	ti(TimeSinceLaying):IDW85K	3.79	3.97	2.44	0.049
	ti(TimeSinceLaying):IDW77K	2.15	2.62	3.33	0.021
	ti(TimeSinceLaying):IDW72K	3.77	3.97	8.74	< 0.001
	ti(TimeSinceLaying):IDW71K	1.00	1.01	2.32	0.13
	ti(TimeSinceLaying):IDW65E	0.00	0.00	0.29	0.99
	ti(TimeSinceLaying):IDW60E	1.00	1.00	6.03	0.014
	ti(TimeSinceLaying):IDW58K	2.37	2.87	3.45	0.012
	ti(TimeSinceLaying):IDW57E	2.02	2.48	3.75	0.020
	ti(TimeSinceLaying):IDW52K	1.00	1.00	9.11	0.003
	ti(TimeSinceLaying):IDW46E	1.00	1.00	0.80	0.37
	ti(TimeSinceLaying):IDW30K	1.00	1.00	1.93	0.16
	ti(TimeSinceLaying):IDW29E	2.26	2.73	6.56	< 0.001
	ti(TimeSinceLaying):IDW26K	1.00	1.00	0.45	0.50
	ti(TimeSinceLaying):IDW25K	3.65	3.93	1.94	0.17
	ti(TimeSinceLaying):IDW20E	1.01	1.02	0.03	0.88
	ti(TimeSinceLaying):IDW14K	1.34	1.60	4.80	0.033
	ti(TimeSinceLaying):IDW12K	3.14	3.61	2.65	0.027
	ti(TimeSinceLaying):IDW05K	3.68	3.93	4.48	0.005
	ti(TimeSinceLaying):IDW04E	3.34	3.76	4.19	0.002
	ti(TimeSinceLaying):IDW02E	2.61	3.12	5.97	< 0.001
	ti(TimeSinceLaying):IDR94A	2.01	2.47	10.70	< 0.001
	ti(TimeSinceLaying):IDR91A	1.00	1.00	5.36	0.021
	ti(TimeSinceLaying):IDR85C	3.86	3.99	4.96	0.001
	ti(TimeSinceLaying):IDR72A	2.46	2.96	8.80	< 0.001
	ti(TimeSinceLaying):IDR64C	1.00	1.00	1.18	0.28

ti(TimeSinceLaying):IDR63C	1.00	1.00	5.69	0.017
ti(TimeSinceLaying):IDR62C	3.72	3.95	4.55	0.001
ti(TimeSinceLaying):IDR62A	1.00	1.00	12.15	< 0.001
ti(TimeSinceLaying):IDR61A	3.07	3.54	5.99	< 0.001
ti(TimeSinceLaying):IDR60A	0.00	0.01	0.37	0.96
ti(TimeSinceLaying):IDR59C	3.45	3.83	3.66	0.004
ti(TimeSinceLaying):IDR54A	1.00	1.00	0.03	0.85
ti(TimeSinceLaying):IDR53A	1.00	1.00	1.99	0.16
ti(TimeSinceLaying):IDR49C	3.84	3.98	3.42	0.009
ti(TimeSinceLaying):IDR46C	1.63	2.01	2.16	0.11
ti(TimeSinceLaying):IDR44C	2.40	2.90	7.79	< 0.001
ti(TimeSinceLaying):IDR40C	3.38	3.79	4.52	< 0.001
ti(TimeSinceLaying):IDR39C	2.01	2.47	1.66	0.27
ti(TimeSinceLaying):IDR37A	1.00	1.00	0.32	0.58
ti(TimeSinceLaying):IDR30A	3.80	3.97	2.82	0.029
ti(TimeSinceLaying):IDR23A	2.39	2.88	4.14	0.006
ti(TimeSinceLaying):IDR14A	3.49	3.85	4.50	0.005
ti(TimeSinceLaying):IDR08A	2.70	3.21	3.06	0.022
ti(TimeSinceLaying):IDR02A	1.00	1.00	5.59	0.018
ti(TimeSinceLaying):IDR01C	1.83	2.25	4.57	0.007
ti(TimeSinceLaying):IDB68P	3.20	3.66	0.96	0.28
ti(TimeSinceLaying):IDB65P	3.81	3.98	4.83	0.002
ti(TimeSinceLaying):IDB52P	3.66	3.93	5.53	< 0.001
ti(TimeSinceLaying):IDB30P	3.81	3.97	5.22	< 0.001
ti(TimeSinceLaying):IDB29P	2.77	3.28	0.51	0.58
ti(TimeSinceLaying):IDB19P	1.00	1.01	0.34	0.56
ti(TimeSinceLaying):IDB18P	1.23	1.41	0.04	0.85
ti(TimeSinceLaying):IDB16P	3.69	3.94	4.44	0.003
ti(TimeSinceLaying):IDB14P	1.00	1.00	6.17	0.013
ti(TimeSinceLaying):IDB04P	1.00	1.00	0.24	0.63
s(ID)	53.61	58.00	12.94	< 0.001

Table S3: Observed and randomized differences between breeding status, sexes and their interactions for the distance to the colony and the 6 activities. Randomized differences are shown as mean ± SD. Significant differences are shown in bold, when the 95% confidence interval does not include 0.

	**$Diff_{obs}$**	**$Diff_{rand}$**	**95% confidence interval**
**Distance to the colony (in km)**			
Success - fail	-54	-55 ± 52	-159 ; 47
Male - female	**-135**	**-135 ± 31**	**-192 ; -74**
**Proportion of time spent foraging**			
male.fail - female.fail	0.018	0.01 ± 0.018	-0.018 ; 0.053
**female.success - female.fail**	**0.044**	**0.044 ± 0.013**	**0.017 ; 0.068**
**male.success - female.fail**	**0.034**	**0.033 ± 0.014**	**0.007 ; 0.058**
female.success - male.fail	0.026	0.026 ± 0.015	-0.0067 ; 0.053
male.success - male.fail	0.015	0.016 ± 0.015	-0.013 ; 0.044
male.success - female.success	-0.011	-0.010 ± 0.008	-0.025 ; 0.006
**Success - fail**	**0.029**	**0.029 ± 0.009**	**0.011 ; 0.048**
Male - Female	-0.003	-0.003 ± 0.007	-0.017 ; 0.012
**Darkness – Daylight**	**-0.22**	**-0.22 ± 0.01**	**-0.23 ; -0.21**
**Non-breeding – chick-rearing**	**-0.034**	**-0.034 ± 0.005**	**-0.045 ; -0.024**
**Proportion of time spent flying**			
male.fail - female.fail	0.037	0.037 ± 0.018	-0.022 ; 0.096
female.success - female.fail	0.009	0.009 ± 0.017	-0.028 ; 0.043
male.success - female.fail	0.031	0.031 ± 0.017	-0.005 ; 0.064
female.success - male.fail	-0.028	-0.028 ± 0.025	-0.077 ; 0.019
male.success - male.fail	-0.006	-0.006 ± 0.024	-0.056 ; 0.042
**male.success - female.success**	**0.022**	**0.022 ± 0.009**	**0.004 ; 0.041**

Success - fail	0.003	0.004 ± 0.014	-0.025 ; 0.032
**Male - Female**	**0.0.25**	**0.025 ± 0.008**	**0.009 ; 0.041**
**Darkness – Daylight**	**-0.19**	**-0.19 ± 0.01**	**-0.20 ; -0.18**
Non-breeding – chick-rearing	0.014	0.015 ± 0.011	-0.007 ; 0.035
**Proportion of time spent floating**			
male.fail - female.fail	-0.056	-0.058 ± 0.037	-0.136 ; 0.015
female.success - female.fail	-0.053	-0.053 ± 0.023	-0.101 ; -0.006
male.success - female.fail	-0.065	-0.064 ± 0.023	-0.11 ; -0.016
female.success - male.fail	0.003	0.004 ± 0.031	-0.058 ; 0.065
male.success - male.fail	-0.009	-0.007 ± 0.031	-0.071 ; 0.057
male.success - female.success	-0.012	-0.011 ± 0.010	-0.030 ; 0.010
Success - fail	-0.033	-0.032 ± 0.018	-0.065 ; 0.006
**Male - Female**	**-0.022**	**-0.022 ± 0.011**	**-0.043 ; -0.001**
**Darkness – Daylight**	**0.41**	**0.41 ± 0.01**	**0.40 ; 0.43**
Non-breeding – chick-rearing	0.020	0.020 ± 0.012	-0.004 ; 0.043
